# Manipulation of Morphology, Particle Size of Barium Sulfate and the Interacting Mechanism of Methyl Glycine Diacetic Acid

**DOI:** 10.3390/molecules28020726

**Published:** 2023-01-11

**Authors:** Jing Li, Yanan Zhou, Jingkang Wang, Na Wang, Jingtao Bi, Xin Li, Kui Chen, Hongxun Hao

**Affiliations:** 1 College of Chemical Engineering, North China University of Science and Technology, Tangshan 063210, China; 2 National Engineering Research Center of Industrial Crystallization Technology, School of Chemical Engineering and Technology, Tianjin University, Tianjin 300072, China; 3 Beijing Institute of Biological Products Co., Ltd., Beijing 100176, China

**Keywords:** barium sulfate, morphology, MGDA, particle size, binding energy

## Abstract

In this paper, methyl glycine diacetic acid (MGDA) was found to have great influence on the morphology and particle size of barium sulfate. The effects of additive, concentration, value of pH and reaction temperature on the morphology and particle size of barium sulfate were studied in detail. The results show that the concentration of reactant and temperature have little effect on the particle size of barium sulfate. However, the pH conditions of the solution and the dosage of MGDA can apparently affect the particle size distribution of barium sulfate. The particle size of barium sulfate particles increases and the morphology changes from polyhedral to rice-shaped with the decreasing of the dosage of MGDA. In solution with higher pH, smaller and rice-shaped barium sulfate was obtained. To investigate the interacting mechanism of MGDA, the binding energy between MGDA and barium sulfate surface was calculated. It was found that the larger absolute value of the binding energy would result in stronger growth inhibition on the crystal face. Finally, the experimental data and theoretical calculations were combined to elucidate the interacting mechanism of the additive on the morphology and particle size of barium sulfate.

## 1. Introduction 

Barium sulfate is known as an important inorganic salt, which has shown widespread applications in industries such as medicine, paint, rubber and papermaking [1,2,3]. In the paint industry, barium sulfate is frequently used as filler for many kinds of coating due to its high whiteness, low oil absorption and great hiding power. As for rubber and plastics industries, using barium sulfate as filler can greatly improve the overall performance of rubber, such as changing the toughness and strength, enhancing the mechanical properties. The thermal property of resin can be significantly improved by adding barium sulfate since barium sulfate has excellent compatibility with resin [4]. It is also quite common to use barium sulfate as a coated-paper and surface-coating agent of photographic paper in the paper industry owing to its low cost. What is more, using barium sulfate with a great oil absorption value could enhance the ink receptivity of paper.

The properties and applications of barium sulfate are closely related to its particle size and morphology. Barium sulfate with average particle size less than 0.5 μm can be used as filler of polyester fiber to improve the processability of polyester fiber [5]. Barium sulfate with particle sizes ranging from 0.8 to 1.0 μm can be applied in coatings, including electrophoretic coatings, plastic coatings, spray powder coatings and high-gloss coatings [6,7]. Barium sulfate with particle sizes less than 10 μm has similar strength, whiteness, oil absorption value and refractive index as titanium dioxide. It can be added into colored pigments as a dispersant to improve the brightness, glossiness, smoothness and fullness of pigments [8]. About 2–20 μm barium sulfate particles play an important role in the manufacture of cosmetics and synthetic resin. In addition, scaly barium sulfate can be used in the papermaking industry because it can optimize the coating performance of paper [9]. Barium sulfate with a high aspect ratio can be applied in the production of rubber and plastics to improve the tensile strength and wear resistance. Spherical barium sulfate can be used in ceramics to enhance mechanical strength [10]. Therefore, it is crucial to control the morphology and particle size of barium sulfate, according to its specific application. Some methods have been reported to control the particle size and morphology of barium sulfate. Jones et al. studied the effects of many kinds of additives, such as ethylenediaminetetraacetic acid (EDTA), nitrilotrimethylenephosphonate (NTMP), N-methylnitrilodimethylenephosphonate (MNDP) and nitrilotriacetic acid (NTA) [11,12,13,14]. However, no general rules have been found, and the molecular mechanism of the effect of additives on the morphology of barium sulfate was not well understood.

In this study, the manipulation of morphology and particle size of barium sulfate was investigated in detail. The effects of additive, concentration, value of pH and reaction temperature on the morphology and particle size of barium sulfate were studied. The effect of six additives were investigated, and methyl glycine diacetic acid (MGDA) was found to have significant effect on the particle size distribution and morphology of barium sulfate. Value of pH could also affect the morphology and particle size of barium sulfate. Furthermore, it has been well known that molecular simulation can facilitate the better understanding of the molecular mechanism of experimental phenomena [15]. Therefore, molecular simulation was carried out to calculate the binding energy between MGDA and each crystal surface to further reveal the molecular mechanism of morphological changes of barium sulfate [16]. These results could provide an important basis for the preparation of barium sulfate with desired morphology and particle size.

## 2. Results and Discussion

### 2.1. Effect of Additives on Morphology and Particle Size of Barium Sulfate

**Figure 1:**
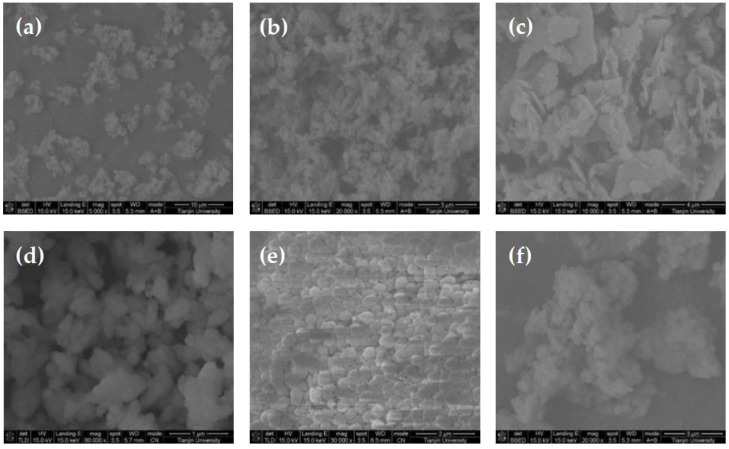
SEM photographs of barium sulfate product obtained in the presence of different additives (reactant concentration 0.1M): (**a**) tetrasodium iminodisuccinate; (**b**) sodium hexametaphosphate; (**c**) PEG400; (**d**) polyaspartic acid; (**e**) MGDA; (**f**) sodium pyrophosphate decahydrate.

**Figure 2:**
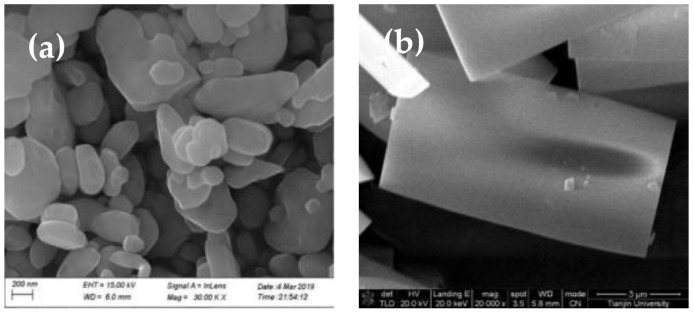
SEM of barium sulfate product obtained without additives: (**a**) reactant concentration 0.1 M; (**b**) reactant concentration 0.005 M.

The effects of six additives (including tetrasodium iminodisuccinate, sodium hexametaphosphate, PEG400, polyaspartic acid, MGDA, sodium pyrophosphate decahydrate) on the morphology and particle size of barium sulfate were investigated. SEM of products obtained with different additives are showed in Figure 1. Figure 2 shows the morphology of barium sulfate without additives. It can be seen that the morphology of barium sulfate changed dramatically when additives were introduced, suggesting that the additives could affect the morphology of barium sulfate. Further analysis shows that crystals of barium sulfate coalesces obviously when the additive was tetrasodium iminodisuccinate, sodium hexametaphosphate or sodium pyrophosphate decahydrate. When the additive was PEG400, the product obtained was flaky, while rice-shaped barium sulfate could be obtained when polyaspartic acid was used as an additive. More importantly, it could be clearly seen that pillow-shaped barium sulfate was obtained and that the morphology homogeneity of barium sulfate was the best when MGDA was used as an additive.

**Figure 3:**
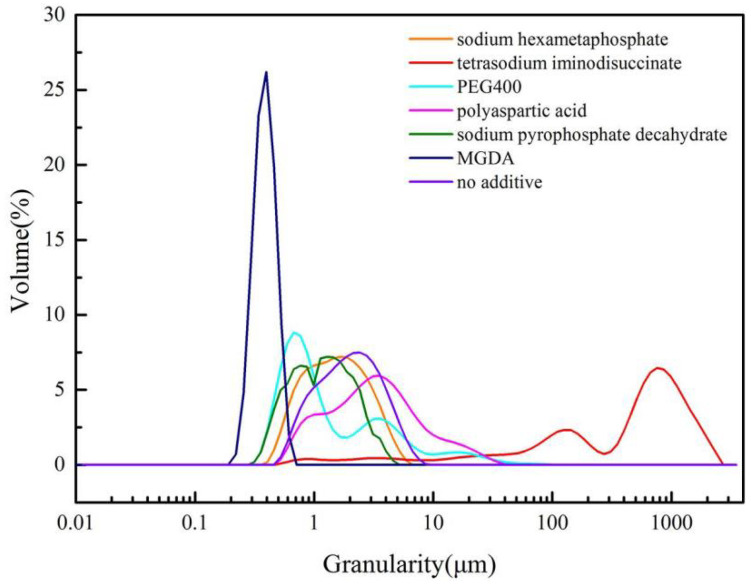
Particle size distribution of barium sulfate products obtained in the presence of different additives.

Figure 3 shows the particle size distribution (PSD) of barium sulfate obtained in the presence of different additives. When MGDA exists, the PSD of barium sulfate is the most uniform, and the particle size is the smallest (average granularity: 381.9 nm). When other additives were introduced, multi-peaks appear in PSD, indicating the decrease in particle size distribution uniformity. Above all, it can be concluded that MGDA could significantly affect the morphology and particle size of barium sulfate.

**Figure 4:**
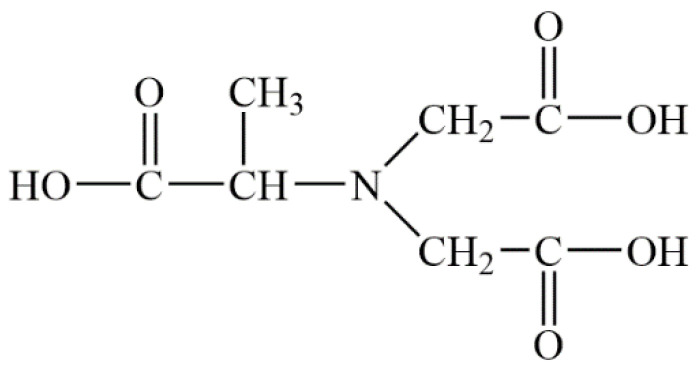
The molecular structure of MGDA.

It has been known that MGDA is a new kind of micromolecule complexing agents, and its molecular structure is shown in Figure 4. MGDA can react with the metal ions and form stable complexes in the range of pH from 2 to 13.5. The complexation constant of MGDA with Ba^2+^ is 4.9 at 25 °C [17]. However, if MGDA only has complexation ability, it can only control the rate of the reaction without affecting the morphology. Previous studies [1,18] have demonstrated that EDTA, DTPA and other complexing agents have dual functions, including complexation with Ba^2+^ to control the reaction process and adsorped onto specific crystal faces of barium sulfate to affect the morphology of barium sulfate. Based on the fact that MGDA is structurally similar to EDTA and DTPA, it is reasonable to speculate that MGDA will adsorb on specific surfaces of barium sulfate, thereby inhibiting their growth and finally changing the morphology of barium sulfate.

### 2.2. Effect of Dosage of MGDA on Morphology and Particle Size of Barium Sulfate

**Figure 5:**
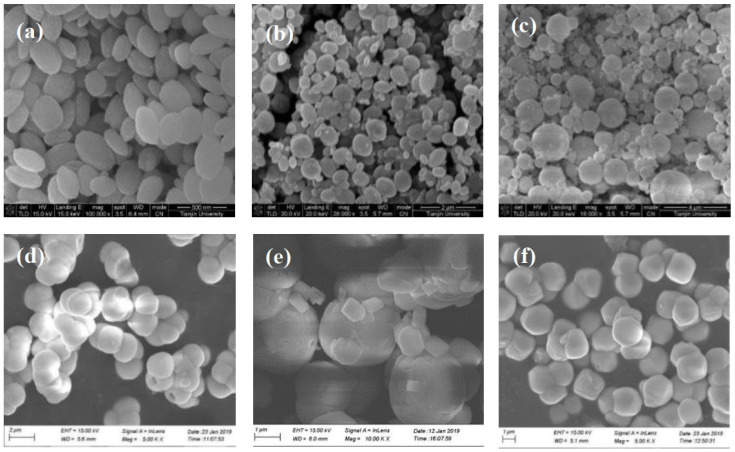
SEM of barium sulfate obtained under different additive dosages. The molar ratio of the additive to barium sulfate is: (**a**) 1:1; (**b**) 1:2; (**c**) 1:4; (**d**) 1:8; (**e**) 1:16; (**f**) 1:20 (reactant concentration 0.005 M.

In order to better understand the effect of MGDA on barium sulfate, the effect of the dosage of MGDA on the morphology and particle size of barium sulfate was further investigated. The reaction conditions and particle sizes of barium sulfate are listed in Table 1. When the dosage of MGDA is decreased, fewer MGDA anions will form complexation with Ba^2+^, and thus the release of barium ion cannot be controlled effectively. As a result, less regulated reaction progress will result in uneven particle size in the solution. On the other hand, the number of MGDA molecules adsorbed on the faces of barium sulfate also decreases, and the inhibition effect on the growth of crystal faces of barium sulfate will decrease, resulting in the changing of the crystal morphology and agglomeration of the particles. As shown in Table 1, when the dosage of additive decreases, the particle size of the product measured by the Malvern particle size analyzer gradually increases, and the uniformity of particle distribution decreases, which is consistent with the theoretical expectation. As shown in Figure 5, the barium sulfate particles gradually coalesce as the dosage of MGDA decreases. This is because MGDA plays a role as an effective dispersant in addition to a complexing agent. When its dosage is reduced, its ability to prevent agglomeration between particles also reduces, eventually leading to the coalescence of barium sulfate particles.

In addition, the SEM of Figure 5 also shows that, when the molar ratio of additive to barium sulfate is 1:20, barium sulfate crystals show multiple crystal planes. With the increase in additive dosage, the particles gradually become spherical, then pillow-shaped and finally rice-shaped. The change in the morphology of barium sulfate crystals with the addition of additives once again indicates that the MGDA molecule can be adsorbed on the specific faces of barium sulfate, inhibiting the growth of the crystal face, thus changing the relative growth rates between the crystal faces and finally changing the crystal morphology.

**Figure 6:**
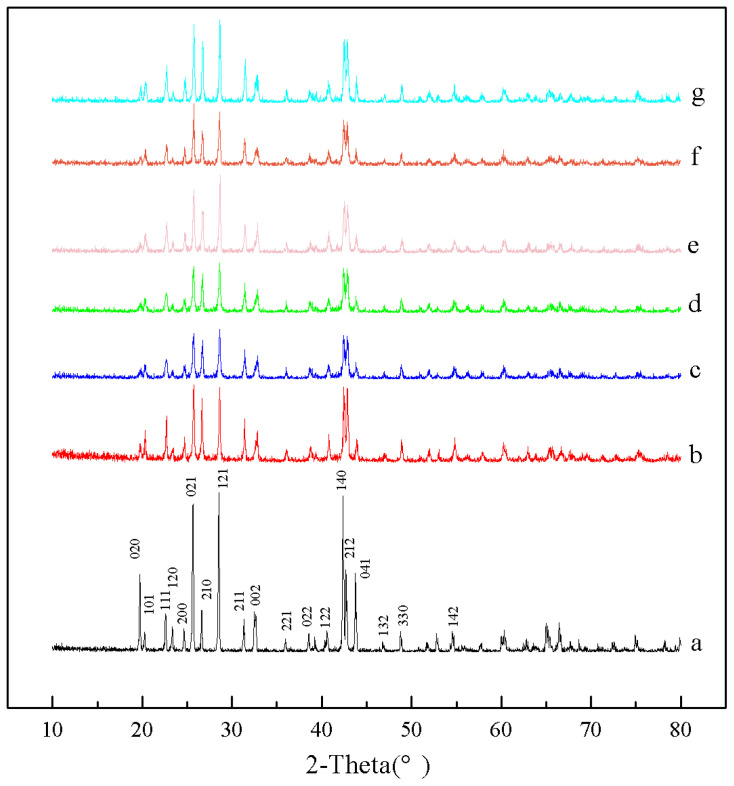
XRD patterns of barium sulfate obtained under different additive dosages, the molar ratio of the MGDA to barium sulfate is: (**a**) no additives (**b**) 1:1 (**c**) 1:2 (**d**) 1:4 (**e**) 1:8 (**f**) 1:16 (**g**) 1:20 (reactant concentration 0.005 M).

The XRD patterns of the barium sulfate products obtained at different dosages of additives are shown in Figure 6. It can be seen that the crystallinities of barium sulfate products obtained in presence of MGDA is lower than those in the absence of MGDA, indicating that MGDA has an inhibitory effect on the growth of barium sulfate. In addition, compared with the barium sulfate XRD patterns without additives, the relative intensities of the diffraction peaks corresponding to the (2 1 2) and (2 1 0) crystal planes are enhanced in the XRD patterns of barium sulfate, indicating that MGDA may interact with these two faces preferentially.

### 2.3. Effect of Concentration of Reactants on the Morphology of Barium Sulfate

**Figure 7:**
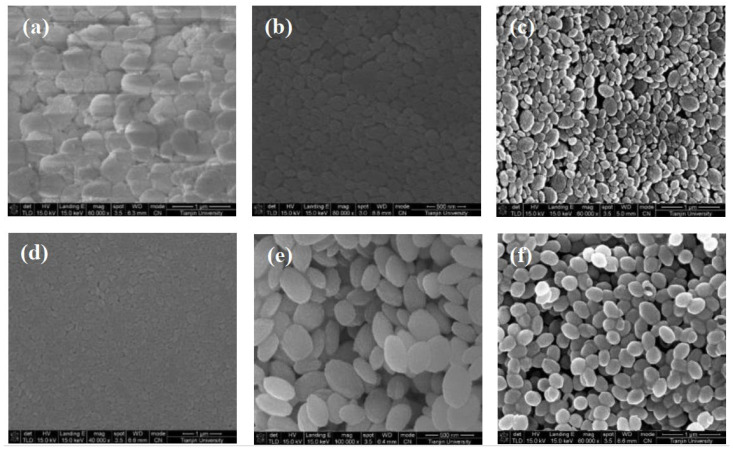
SEM of barium sulfate product obtained at different reactant concentrations at 25 °C: (**a**) 0.1 M; (**b**) 0.05 M; (**c**) 0.04 M; (**d**) 0.025 M; (**e**) 0.005 M; (**f**) 0.001 M.

Concentration is an important factor that could affect crystal growth process [18,19]. Without adjusting the pH, the molar concentration of BaCl_2_ in the reaction solution is 0.1 M, 0.05 M, 0.04 M, 0.025 M, 0.005 M, 0.001 M, respectively, and the molar concentration of MGDA-3Na added is equal to that of barium chloride. Figure 7 shows the morphology of barium sulfate products obtained at different concentrations. It can be seen that the concentration of the reactants does not have significant influence on the morphology of barium sulfate. When the concentration of BaCl_2_ and Na_2_SO_4_ in the solution is 0.1 M and 0.05 M, pillow-shaped barium sulfate was obtained. In the concentration range of 0.04–0.001 M, the morphology of barium sulfate transforms from a pillow shape to a rice shape. In addition, it can be noticed that both the particle size distribution and morphology of the product are more uniform when the concentration of the reactants is lower. It can be speculated that the particles are better mixed in the solution under low concentration and therefore result in more uniform morphology and better particle dispersibility of the product.

**Figure 8:**
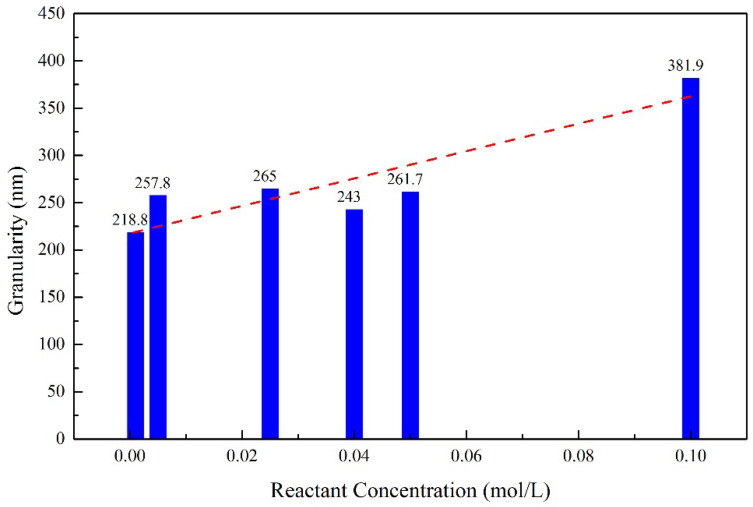
Relationship between reactant concentration and barium sulfate particle size.

Figure 8 shows the variation of the particle size of barium sulfate with the concentration of the reactants. The particle size of the product increases as the concentration of the reactants increases. It is well known that the particle size and morphology of crystal are determined by the nucleation, growth and agglomeration of crystals [20,21,22,23]. When the concentration of the solution is low, there is little chance of collision between the particles, and thus it is difficult for the newly formed crystal nuclei to aggregate into large particles. When the concentration of the solution increases, both the nucleation rate and the chance of collision between the particles will increase. Therefore, the particles tend to coalesce and form bigger particles. The particle size of barium sulfate obtained in these experiments are in the range of 218.8 nm to 381.9 nm.

### 2.4. Effect of pH Value on the Morphology of Barium Sulfate

The MGDA molecule that has different ionization balance in aqueous solution is a zwitterion. MGDA can act as a complexing agent. The protonated MGDA anion can combine with Ba^2+^ to form a stable complex, thus controlling the progress of the precipitation reaction. Figure S1 of the Supporting Information shows the SEM images of barium sulfate products at natural pH and other pH conditions with reactant concentrations of 0.1 M, 0.005 M and 0.001 M, respectively. It was found that at the same reactant concentration, when the pH of the reactant is around 7 or 9, the product particles are uniform and the dispersibility is good. As the pH value decreases, the morphology of barium sulfate gradually becomes less regular, and the dispersibility also decreases.

**Figure 9:**
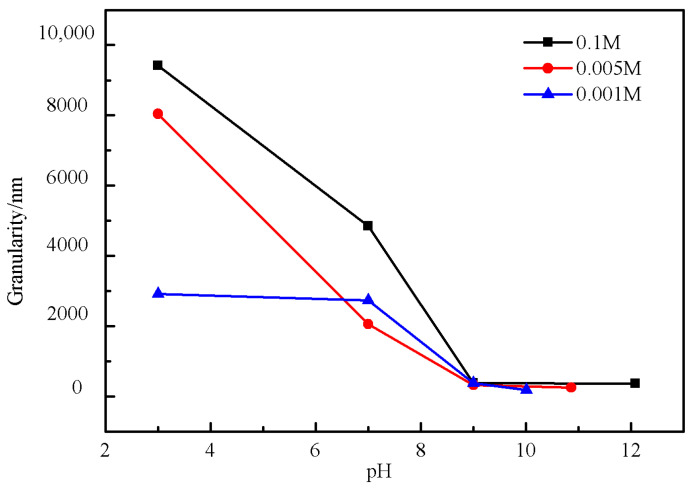
Relationship between pH and product particle size under different reactant concentrations.

**Figure 10:**
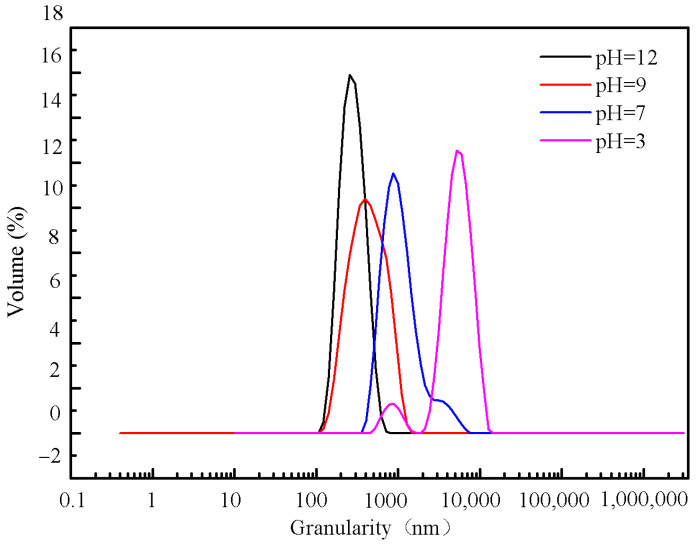
Product PSD obtained under different pH conditions (reactant concentration 0.005 M).

Figure 9 shows that the particle size of barium sulfate increases with the decreasing of the pH under the same reactant concentration. Besides, the particle size is minimized when the solution is around 7. The particle size distributions of the products obtained at different pH values when the concentration of the reactant is 0.005 M are shown in Figure 10. It can be seen that the PSD curve has a side peak at pH = 7 and a double peak at pH = 3, respectively. In contrast, the PSD is uniform at both pH = 9 and pH = 12, which was verified by the corresponding single peak. What is more, the size distribution at pH = 12 is narrower and more symmetrical than that at pH = 9. Therefore, it can be concluded that the larger the pH value is, the more uniform the PSD of the product will be. It indicates that MGDA-3Na has a more positive effect on the uniformity of PSD under higher pH conditions. In a basic environment, MGDA is mostly in the form of anions, which can form a complex with Ba^2+^. While at lower pH, MGDA anions tend to combine with the hydrogen protons, and thus the number of MGDA anions available for complexation with barium ions is reduced. As a result, the number of free barium ions in the solution increases, leading to a higher supersaturation degree. Furthermore, the reaction cannot be well controlled since the solution is relatively concentrated. Therefore, the particles of the BaSO_4_ product are prone to coalesce at lower pH, resulting in larger particles and less uniform PSD.

MGDA is one kind of metal complexing agent that can form stable complexes with metal ions in solution. The parameter that characterizes its complexation ability is called the stability constant *K*. The higher the value of *K* is, the stronger the complexation ability of MGDA is and the more stable the as-formed complex will be. The complexing reaction can be expressed as follows (the charge is omitted):

M + L = ML(1)

where M represents a metal ion, and L stands for a MGDA anion. The stability constant can be expressed as Equation (2):

(2) $$K_{\mathrm{ML}}=\frac{\left[\mathrm{ML}\right]}{\left[M\right]\left[L\right]}$$

Previous research has pointed out that the stability constant of MGDA and Ba^2+^ is 4.9 at 25 °C, demonstrating the good complexing ability of MGDA [12]. In terms of structure, MGDA can be classified as an aminocarboxylate complexing agent due to the existence of three carboxylic acid groups and one amphoteric nitrogen atom. When the acidity of the solution is enhanced, each of the nitrogen atom and the carboxylic acid groups may accept a hydrogen proton and finally form H_4_L^+^. Conversely, under alkaline conditions, deprotonation is more predominant. The ionization equilibrium in the solution can be explained as follows:


(3) $$H_{4}L^{+}\overset{-H^{+}}{\underset{+H^{+}}{⇄}}H_{3}L\overset{-H^{+}}{\underset{+H^{+}}{⇄}}H_{2}L^{-}\overset{-H^{+}}{\underset{+H^{+}}{⇄}}HL^{2-}\overset{-H^{+}}{\underset{+H^{+}}{⇄}}L^{3-}$$

The ionization equilibrium changes with the variations of hydrogen ions concentration (pH). As a result, the complexing ability of MGDA will vary with pH. This phenomenon is called the acid effect. The magnitude of the acid effect is usually expressed by the acid effect coefficient, which is the ratio between the total concentration of various forms of MGDA that cannot participate in the complexation reaction and the equilibrium concentration of L^3−^ that can participate in the complexation reaction. The acid effect coefficient(*α*_L[H]_) can be calculated as:

(4) $$\begin{array}{cc} \alpha_{L[H]} & =\frac{\left[L\prime \right]}{\left[L^{3-}\right]}=\frac{{[L}^{3-}{]+[\mathrm{HL}}^{2-}{]+[H}_{2}L^{-}{]+[H}_{3}{L]+[H}_{4}L^{+}]}{{[L}^{3-}]}\\ & =1+\frac{{[H}^{+}]}{K_{4}}+\frac{{\left[H^{+}\right]}^{2}}{K_{4}K_{3}}+\frac{{\left[H^{+}\right]}^{3}}{K_{4}K_{3}K_{2}}+\frac{{\left[H^{+}\right]}^{4}}{K_{4}K_{3}K_{2}K_{1}} \end{array}$$

where *K*_n_ is the equilibrium dissociation coefficient, and [H^+^] represents the hydrogen ion concentration. It can be concluded that, as the pH increases, the acid effect coefficient *α*_L(H)_ decreases, since [H]^+^ decreases and all *K*_n_ are constants.

Combining Equations (2) and (4), a new equation can be obtained as follows:

(5) $$\frac{\left[\mathrm{ML}\right]}{\left[M\right]\left[L^{\prime }\right]}=\frac{K_{\mathrm{ML}}}{\alpha_{L(H)}}=K_{\mathrm{ML}}^{\prime }$$

where $K_{MY}^{\prime }$ is called the conditional stability constant that considers the acid effect and thus can describe the complexing ability of MGDA under experimental conditions. It can be seen from Formulas (4) and (5) that the *α*_L(H)_ decreases as the pH increases, resulting in an increase in the conditional stability constant. Therefore, at higher pH, MGDA can better control the progress of the reaction, resulting in more uniform product particles with better particle size dispersion. This prediction based on theoretical calculation is consistent with the experimental results.

**Figure 11:**
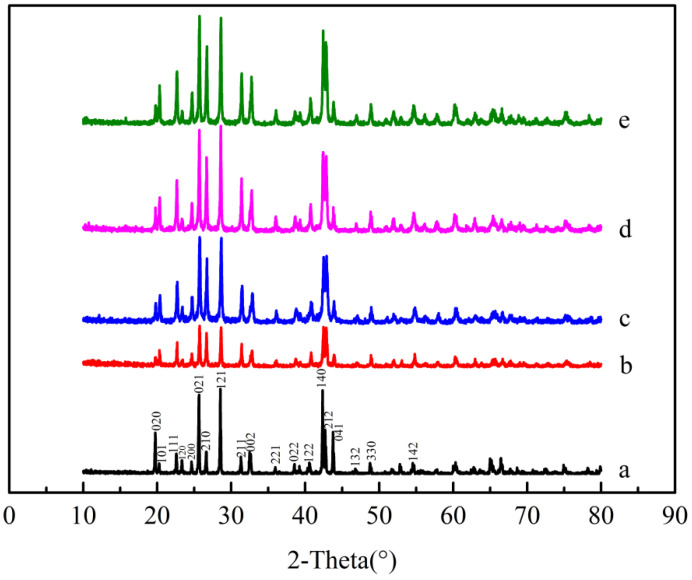
Standard XRD pattern of barium sulfate and XRD patterns of barium sulfate products obtained at different pH (reactant concentration 0.005 M): (**a**) standard pattern; (**b**) natural pH; (**c**) pH = 9; (**d**) pH = 7; (**e**) pH = 3.

The barium sulfate products obtained under different pH values at a reactant concentration of 0.005 M were analyzed by X-ray diffraction (XRD), and the results are given in Figure 11.

Comparing with the standard pattern, it can be found that no polymorphism phenomenon was observed under different pH conditions [24]. However, as the pH decreases, the crystallinity of the product gradually increases, indicating that MGDA has a strong inhibitory effect on the surface of barium sulfate at high pH and a weak inhibitory effect at low pH. This is because MGDA is deprotonated at higher pH, and deprotonated MGDA is more likely to interact with the surfaces of barium sulfate crystals, inhibiting crystal surface growth and reducing the crystallinity. The change in the relative intensity of each peak in the XRD pattern can indicate the variation in the crystal surface. Comparing the XRD patterns of barium sulfate products obtained under different pH conditions with the standard pattern of barium sulfate at a concentration of 0.005 M, it can be found that the relative diffraction intensities of (2 1 1), (2 1 0), (2 1 2) surfaces change greatly with pH. Therefore, it can be suspected that MGDA has specific adsorption effects on the (2 1 1), (2 1 0), (2 1 2) faces of BaSO_4_ crystals and can significantly affect the growth rates of these faces. Therefore, the MGDA added into the reaction mother liquors will result in changes of the morphology of barium sulfate.

### 2.5. Effect of Temperature on the Morphology and Particle Size of Barium Sulfate

**Figure 12:**
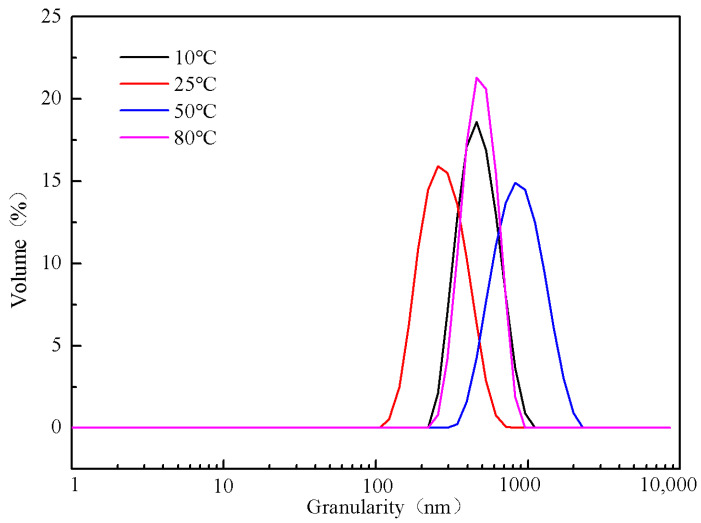
PSD of barium sulfate obtained at different reaction temperatures (reactant concentration 0.005 M).

The particle size and morphology of barium sulfate at 10 °C, 25 °C, 50 °C and 80 °C were also investigated when MGDA is present at a concentration of 0.005 M. The results are given in Figure 12 and Figure S2 of the Supporting Information. It can be seen from Figure 12 that the average particle size of barium sulfate obtained at 10 °C, 25 °C, 50 °C and 80 °C are 457.6 nm, 257.8 nm, 795.2 nm and 446.2 nm, respectively. At 25 °C, the product has the smallest particle size. The barium sulfate product with uniform PSD and good dispersibility can be obtained at any temperature, which means that the reaction temperature has a relatively small influence on the dispersion degree of barium sulfate particles. The barium sulfate with uniform PSD can be obtained at any temperature. It can be seen from Figure S2 that the morphologies of the products obtained at different temperatures are almost the same (rice-shaped).

### 2.6. Molecular Mechanism of the Effect of MGDA on the Morphology and Particle Size of Barium Sulfate

**Figure 13:**
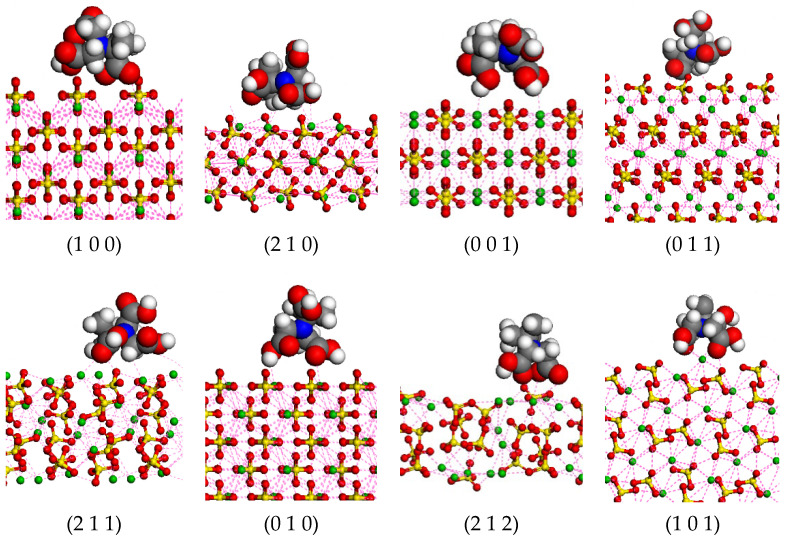
Interactions between MGDA and each crystal surface of barium sulfate.

In order to better understand the interactions between MGDA and barium sulfate, an interface model was constructed according to the molar ratio of MGDA to barium sulfate at 1:1. Molecular dynamics simulation was carried out to calculate the binding energy between each crystal surface of barium sulfate and MGDA. Figure 13 shows the interactions between MGDA and each surface. The pink dotted line indicates the interactions between the atoms. It can be seen that the MGDA molecular conformation varies with the crystal face of BaSO_4_. However, the carboxylic acid groups in the MGDA molecule are all close to the barium sulfate surface. It can be seen clearly that, on the (0 0 1) and (1 0 1) faces, the three carboxylic acid groups of the MGDA molecule are oriented toward the barium sulfate surfaces, indicating that the carboxylic acid group is the main functional group that causes the interactions between MGDA and barium sulfate. What is more, carboxylic acid groups interact with barium ions by electrostatic force. Except for carboxylic acid groups, there is also certain repulsion between the two methyl groups in the carbon bone chain of MGDA molecule and crystal surfaces of BaSO_4_, as implied in Figure 13. The interactions between MGDA and barium sulfate are similar to those between EDTA and barium sulfate because both MGDA and EDTA are aminocarboxylate complexing agents [3,14]. It has been reported that the complexation constant of MGDA with barium ions is 4.9, while the complexation constant of EDTA with barium ions is 7.76 [25]. Generally, the larger the complex constant is, the more stable the complex will be. The EDTA molecule has one more carboxylic acid group and a longer carbon bone chain than the GDA molecule. Thus, EDTA is more prone to twist, allowing carboxylic acid groups to interact with barium sulfate more easily. In addition, compared with MGDA, EDTA does not contain methyl groups that are repulsive to the crystal surfaces of BaSO_4_. For these reasons, EDTA molecules are more likely to interact with barium sulfate. The existence of carboxylic acid groups in additives is beneficial to strengthen the interactions between additives and barium sulfate faces, while methyl groups have the opposite effect. These conclusions are of great significance in the design of additives for manipulating the morphology and particle size of barium sulfate.

**Figure 14:**
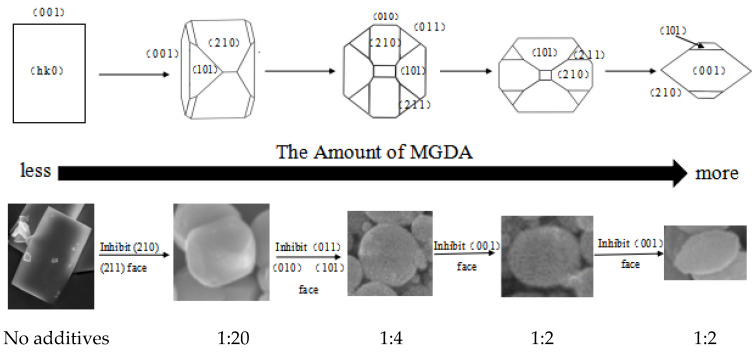
The evolution of barium sulfate morphology with different molar ratio of MGDA-3Na to BaCl_2_.

The binding energies calculated are listed in Table 2. Generally, the greater the absolute value of the binding energy is, the stronger the affinity between the additive and the crystal face will be. The order of the absolute values of the binding energies turns out to be: (2 1 2) > (2 1 0) > (2 1 1) > (1 0 0) > (0 1 1) > (0 1 0) > (1 0 1) > (0 0 1). Jones et al. calculated the attachment energy with relaxation and listed the faces according to the absolute value of the attachment energy: (2 1 2) > (0 1 0) > (0 1 1) > (2 1 1) > (1 0 0) > (2 1 0) > (1 0 1) > (0 0 1). The smaller the absolute value of the attachment energy is, the larger the area of the crystal surface will be. The absolute value of the binding energy of (2 1 2) face is the largest, meaning that the affinity of MGDA to the (2 1 2) face is the strongest. However, since the initial attachment energy of the (2 1 2) face is too large, the growth rate of the (2 1 2) face is still fast and the (2 1 2) face is difficult to see even if MGDA has a strong inhibitory effect on the crystal surface. It can be seen that the next energetically favored faces are the (2 1 0) and (2 1 1) faces. The XRD patterns in Figure 11 shows that the relative strength of the two peaks representing (2 1 2) and (2 1 0) faces changed significantly, meaning that MGDA is most likely to interact with these two surfaces, which is consistent with the calculation results of binding energy. According to the morphology variation of barium sulfate with different MGDA dosages in Figure 5 and the binding energy calculated in this section, the evolution of barium sulfate morphology in the presence of MGDA is illustrated by Figure 14.

When the dosage of MGDA is low, it first interacts with the (2 1 0) and (2 1 1) faces, and the growth rates of the two faces are slowed down, and the faces are exposed. As the dosage of additive increases, MDGA gradually begins to inhibit the growth of the (0 1 1), (0 1 0), (1 0 1) faces, which are less favorable. The initial attachment energy of these three faces can be listed as: (0 1 0) > (0 1 1) > (1 0 1). MGDA first inhibits the surface, which grows faster, and then the slower one. This is beneficial to reduce the differences in the growth rate between the surfaces, and therefore, the crystal morphology tends to be spherical. Later, the growth of crystal plane (001) is inhibited, and, as a result, the spherical particles become flat. In this case, the area of the (211) face is larger, which makes the two ends of the crystal sharper and tend to be rice-shaped when viewed from the side. With the further increase in the dosage of additive, the proportion of rice granular crystals increases due to the inhibition effect of MGDA towards the growth of the (1 0 0) surface, and finally, rice-shaped crystals will be obtained.

## 3. Experimental

### 3.1. Materials

MGDA-3Na was supplied by TCI Chemical Industry Development Co., Ltd., Shanghai, China. Tetrasodium iminodisuccinate and polyaspartic acid were provided by Think-Do Environment Co., Ltd., Hebei, China. Sodium hexametaphosphate and sodium pyrophosphate decahydrate were supplied by Chemart Chemical Technology Co., Ltd., Tianjin, China. PEG400 was supplied by Jiangtian Chemical Co. Ltd., Tianjin, China. BaCl_2_ (mass concentration 99.5%), and Na_2_SO_4_ (mass concentration 99.5%) was provided by Sailboat Chemical Reagent Technology Co., Ltd., Tianjin, China. Hydrochloric acid (mass concentration 36.0–38.0%) and sodium hydroxide (mass concentration 96.0%) were provided by Yuanli Chemical Co., Ltd., Tianjin, China. All materials were used without further purification.

### 3.2. Crystallization Experiment of Barium Sulfate

Under agitation (300–350 r/min), a certain concentration of BaCl_2_ stock solution (0.001–0.1 M) was placed into a crystallizer, and the solution temperature was controlled at the specified temperature by a thermostat (CF41, Julabo, Germany) with an accuracy of ±0.05 K. A certain amount of additive was added to the crystallizer and was completely dissolved. A Na_2_SO_4_ stock solution of the same molar concentration with BaCl_2_ was added dropwise to the crystallizer using a peristaltic pump at a speed of 1–5 mL/min. During the reaction, the pH of the reaction solution was adjusted to the desired pH with HCl or NaOH solution. After the addition of Na_2_SO_4_ stock solution, the rotation speed was lowered to 150 r/min, and the precipitation was kept aging for 3 h. At last, the suspension was centrifuged, washed twice with ultrapure water and dried at 35 °C for 6 h.

### 3.3. Characterization of Barium Sulfate

#### 3.3.1. Scanning Electron Microscopy (SEM)

The obtained products were placed on carbon-coated SEM stubs and then were sputter-coated with a layer of gold to make the product conductive. The morphology of the barium sulfate was investigated using a scanning electron microscope (S-4800, Hitachi, Japan).

#### 3.3.2. X-ray Powder Diffraction

A Rigaku D/max-2500 X-ray powder diffractometer (Rigaku, Japan, Cu Kα 1.5405 Å) was utilized for the polymorphic characterization of barium sulfate (using a step size of 0.02°, scanning rate of 0.067°/s, diffraction angle (2θ) range of 5 to 80°).

#### 3.3.3. Malvern Zetasizer Nano ZS

The particle size of barium sulfate was studied using dynamic light scattering (Malvern Zetasizer Nano ZS, Malvern Instruments Ltd., Worcestershire, UK), equipped with a He–Ne laser lamp (0.4 mW) with a wavelength of 633 nm. During the measurements, water was used as the dispersed phase, and the samples were constantly stirred at room temperature.

### 3.4. Molecular Simulation

The interaction energy between MGDA and the surface of barium sulfate was calculated through Materials Studio 8 (Accelrys Inc., SanDiego, CA, USA) in order to get a better insight of the growth inhibition effect of MGDA molecules on the surface of barium sulfate. The surface docking model is often used to investigate the interactions between additives and the surface of crystal. In this model, the molecular dynamics are performed based on the assumption that the additive is on the surface of the crystal, and then the binding energy between the additive and the surface can be calculated. The greater the absolute value of the binding energy is, the stronger the interaction between the additive and the surface of barium sulfate will be, which means that the additive will have a more inhibitory effect on the surface [26,27]. This model assumes that the crystal growth can be disrupted by the additive through adsorbing to the specific surface of barium sulfate and thus the morphology of barium sulfate will be changed. The binding energy between MGDA and the cleaved faces can be obtained using molecular simulation.

Ryabov et al. revealed the single-crystal-structure data of barium sulfate in 1983 table [28]. Jones et al. have calculated the initial attachment energy of each surface of barium sulfate under vacuum conditions, and the main surfaces can be listed according to the energy as: (2 1 2) > (0 1 0) > (0 1 1) > (2 1 1) > (1 0 0) > (2 1 0) > (1 0 1) > (0 0 1). The smaller the absolute value of the attachment energy is, the larger the crystal face area will be. In this work, the binding energy between MGDA and these main crystal faces were calculated. Firstly, the geometrical optimization of the structure of barium sulfate single crystal was carried out using universal force field. Secondly, the specific face was cleaved, and a supercell structure was created with appropriate dimensions (U = 3, V = 3). Then, the supercell was spread into a vacuum slab, and a MGDA molecular layer (including 108 MGDA molecules) was created. Through building the vacuum slab and the optimized MGDA molecular layer together, geometrical optimization was carried out to identify the lowest energy conformation. Finally, molecular dynamics simulations were carried out. NVT ensemble was used at a time step of 0.1 fs for 500 ps. The temperature was set at 298 K, and a Nose–Hoover–Langevin thermostat was used to control the temperature during simulation. After the dynamics simulation, the energy module was used to calculate the energy of each part. The binding energy can be calculated as the following equation [29,30]:

(6) $$E_{\mathrm{bind}}=E_{\mathrm{tot}}-(E_{\mathrm{sur}}+E_{\mathrm{add}})$$

where *E*_bind_ represents the binding energy between MGDA and the crystal surface, *E*_tot_ is the energy of the system that docks the additive molecular layer after the molecular dynamics simulation, *E*_surf_ represents the energy of the crystal surface, and *E*_add_ is the energy of the additive layer. The calculated binding energy can be used to interpret the interactions between MGDA and crystal faces.

## 4. Conclusions

In this work, the manipulation of the morphology and particle size of barium sulfate was investigated in detail. The effects of six additives were investigated, and MGDA was found to be able to significantly affect the morphology and particle size of barium sulfate. The effects of the dosage of additive, the concentration of the reactant, the pH value and the reaction temperature on the morphology and particle size of barium sulfate were also investigated. The results show that the concentration of reactant and temperature have little effect on the particle size of barium sulfate. The pH conditions of the solution and the dosage of MGDA can apparently affect the particle size distribution of barium sulfate. The particle size distribution of the product is uniform, and the particle size is less than 1 μm at natural pH. With the decrease in the dosage of MGDA, the particle size of barium sulfate particles increases and the morphology changes from polyhedron to rice-shaped. The interacting mechanism of MGDA was also investigated. It was found that MGDA can not only control the reaction process by complexing with barium ions but also change the morphology of barium sulfate by adsorbing on the specific crystal surfaces. The XRD patterns show that the crystal faces that are preferentially adsorbed by MGDA are (2 1 1), (2 1 0) and (2 1 2). From molecular simulation, it was found that the order of the absolute value of the binding energy of each crystal face is: (2 1 2) > (2 1 0) > (2 1 1) > (1 0 0) > (0 1 1) > (0 1 0) > (1 0 1) > (0 0 1), indicating that (2 1 1), (2 1 0), (2 1 2) faces are more energy-favorable, which is consistent with the XRD characterization results. The greater absolute value of the binding energy will result in a stronger interaction between MGDA and the crystal surface. The existence of a carboxyl group in the MGDA molecule is beneficial to enhance the interaction between MGDA and the crystal surface, while the existence of a methyl group has the opposite effect.

## Figures and Tables

**Table 1:** Effect of the amount of MGDA on the particle size of barium sulfate.

Temperature/°C	Reactant Concentration/M	pH	Molar Ratio of MGDA-3Na to BaCl_2_	BaSO_4_ Granularity/nm
25	0.005	Unregulated	1:1	257.8
25	0.005	Unregulated	1:2	355.7
25	0.005	Unregulated	1:4	304.5
25	0.005	Unregulated	1:8	5486
25	0.005	Unregulated	1:16	5970
25	0.005	Unregulated	1:20	6142

**Table 2:** Binding energies between each crystal face and additive.

Surface	*E*_total_ (kJ/mol)	*E*_surface_ (kJ/mol)	*E*_additive_ (kJ/mol)	*E*_binding_ (kJ/mol)
(1 0 0)	67,506.1	49,569.6	18,390.6	−454.061
(2 1 0)	72,417.5	54,540.5	18,525.1	−648.094
(0 0 1)	71,645.9	53,768.1	18,225.6	−347.824
(0 1 1)	64,920.5	46,853.4	18,500.8	−433.714
(2 1 1)	67,451.8	49,253.3	18,709.2	−510.655
(0 1 0)	69,131.4	51,511.8	18,017.3	−397.643
(2 1 2)	64,630.9	46,467.2	18,861.1	−697.406
(1 0 1)	69,322.4	51,262.5	18,429.1	−369.121

## Data Availability

Not applicable.

## Supplementary Materials

The following supporting information can be downloaded at: https://www.mdpi.com/article/10.3390/molecules28020726/s1. Figure S1. SEM of barium sulfate product obtained with different reactant concentrations at different pH values; Figure S2. SEM of barium sulfate obtained at different reaction temperatures.

## Abbreviations

**Abbreviation**	**Full name**
MGDA	methyl glycine diacetic acid
EDTA	ethylenediaminetetraacetic acid
NTMP	nitrilotrimethylenephosphonate
MNDP	N-methylnitrilodimethylenephosphonate
NTA	nitrilotriacetic acid
SEM	scanning electron microscopy
PSD	particle size distribution
XRD	X-ray diffraction
