# Supplementary material for: Manipulation of Morphology, Particle Size of Barium Sulfate and the Interacting Mechanism of Methyl Glycine Diacetic Acid
Source: Molecules. 2023 Jan 11;28(2):726. doi: 10.3390/molecules28020726 (PMC9863649; doi:10.3390/molecules28020726)
Supplement: Supplementary file 1 [file molecules-28-00726-s001.zip › molecules-2112844-supplementary.pdf]

Supporting Information

Manipulation of Morphology, Particle Size of Barium Sulfate and the Interacting Mechanism of Methyl Glycine Diacetic Acid

Jing Li [1,2,3], Yanan Zhou [1,*], Jingkang Wang [2], Na Wang [2,*], Jingtao Bi [2], Xin Li [2], Kui Chen [2] and Hongxun Hao [2]

1 College of Chemical Engineering, North China University of Science and Technology,
Tangshan 063210, China; llllj@tju.edu.cn

2 National Engineering Research Center of Industrial Crystallization Technology, School of Chemical
Engineering and Technology, Tianjin University, Tianjin 300072, China; jkwang@tju.edu.cn (J.W.);
jingtaob@gmail.com (J.B.); xinlll@tju.edu.cn (X.L.); chenkui@tju.edu.cn (K.C.); hongxunhao@tju.edu.cn (H.H.)

3 Beijing Institute of Biological Products Co., Ltd., Beijing 100176, China

* Correspondence: zynzyn@tju.edu.cn (Y.Z.); wangna224@tju.edu.cn (N.W.)

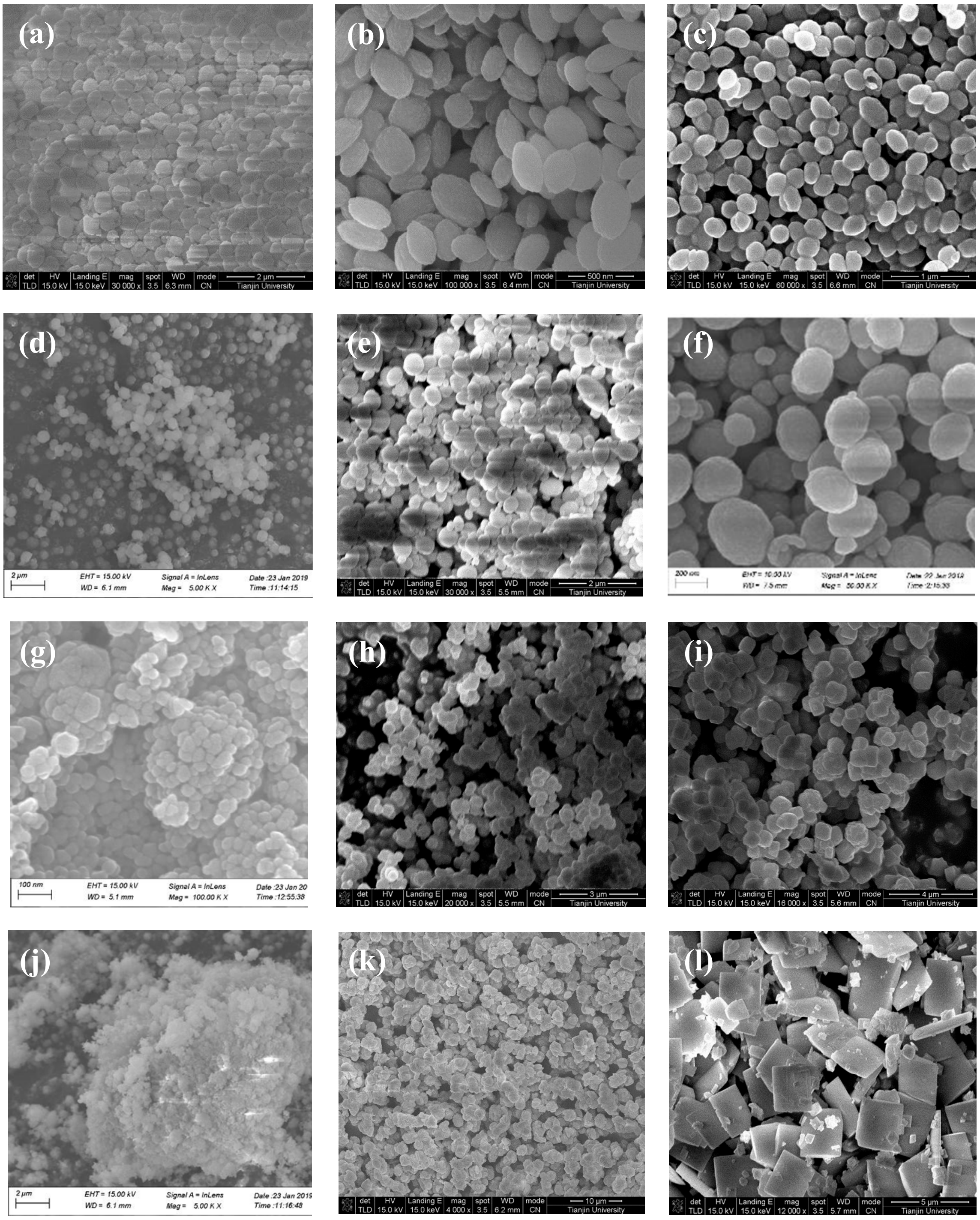

Figure S1. SEM of barium sulfate product obtained with different reactant concentrations at different pH values: (a) 0.1 M, natural pH(pH = 12.0); (b) 0.005 M, natural pH(pH =

10.9); (c) 0.001 M, natural pH(pH = 10.0); (d) 0.1 M, pH = 9.0; (e) 0.005 M, pH = 9.0; (f) 0.001M, pH = 9.0; (g) 0.1 M, pH = 7.0; (h) 0.005 M, pH = 7.0; (i) 0.001 M, pH = 7.0; (j) 0.1 M, pH = 3.0; (k) 0.005 M, pH = 3.0; (l) 0.001 M, pH = 3.0.

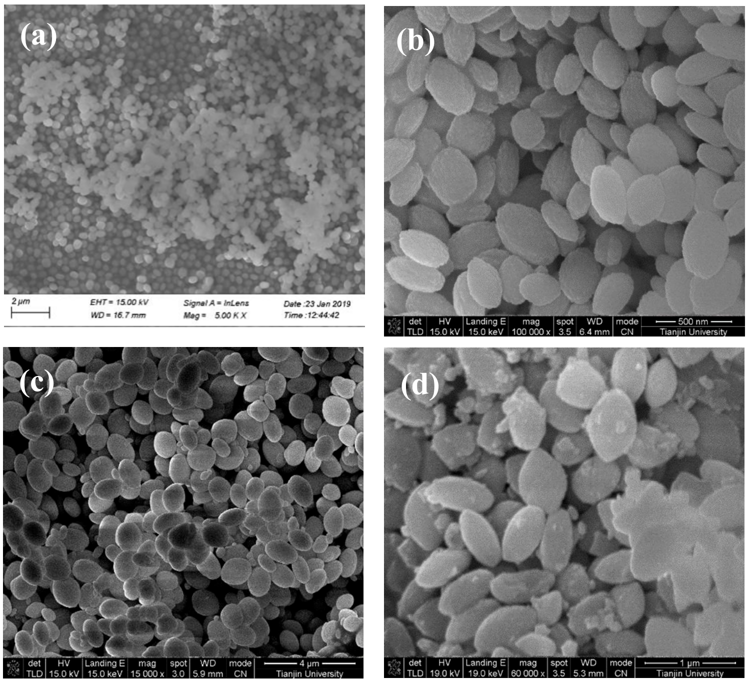

Figure S2. SEM of barium sulfate obtained at different reaction temperatures (reactant concentration 0.005 M): (a) 10 °C; (b) 25 °C; (c) 50 °C; (d) 80 °C.
